# COVID-19 in Solid Organ Transplantation: Results of the National COVID Cohort Collaborative

**DOI:** 10.1097/TXD.0000000000001234

**Published:** 2021-10-06

**Authors:** Amanda J. Vinson, Gaurav Agarwal, Ran Dai, Alfred J. Anzalone, Stephen B. Lee, Evan French, Amy Olex, Vithal Madhira, Roslyn B. Mannon

**Affiliations:** 1 Division of Nephrology, Department of Medicine, Dalhousie University, Halifax, NS, Canada.; 2 Division of Nephrology, Department of Medicine, University of Alabama at Birmingham, AL.; 3 Department of Biostatistics, University of Nebraska Medical Center, Omaha, NE.; 4 Division of Infectious Diseases (Regina), University of Saskatchewan, SK, Canada.; 5 Virginia Commonwealth University, Richmond, VA.; 6 Palila Software, Reno, NV.; 7 Division of Nephology, Department of Medicine, University of Nebraska Medical Center, Omaha, NE.

## Abstract

Coronavirus disease 2019 (COVID-19) has resulted in significant morbidity and mortality in solid organ transplant (SOT) recipients. The National COVID Cohort Collaborative was developed to facilitate analysis of patient-level data for those tested for COVID-19 across the United States.

**Methods.:**

In this study, we identified a cohort of SOT recipients testing positive or negative for COVID-19 (COVID+ and COVID−, respectively) between January 1, 2020, and November 20, 2020. Univariable and multivariable logistic regression were used to determine predictors of a positive result among those tested. Outcomes following COVID-19 diagnosis were also explored.

**Results.:**

Of 18 121 SOT patients tested, 1925 were positive (10.6%). COVID+ SOT patients were more likely to have a kidney transplant and be non-White race. Comorbidities were common in all SOT patients but significantly more common in those who were COVID+. Of COVID+ SOT, 42.9% required hospital admission. COVID+ status was the strongest predictor of acute kidney injury (AKI), rejection, and graft failure in the 90 d after testing. A total of 40.9% of COVID+ SOT experienced a major adverse renal or cardiac event, 16.3% experienced a major adverse cardiac event, 35.3% experienced AKI, and 1.5% experienced graft loss.

**Conclusions.:**

In the largest US cohort of COVID+ SOT recipients to date, we identified patient factors associated with the diagnosis of COVID-19 and outcomes following infection, including a high incidence of major adverse renal or cardiac event and AKI.

## Abstract 2

Supplemental Digital Content is available in the text.

Severe acute respiratory syndrome coronavirus-2 (SARS-CoV-2) infection and the coronavirus disease 2019 (COVID-19) pandemic have resulted in significant morbidity and mortality worldwide.^1^ In the general population, critical manifestations of COVID-19 include an increased risk of acute kidney injury (AKI), major adverse cardiovascular events (MACE), acute respiratory distress syndrome, and death.^2^ The incidence of COVID-19 in solid organ transplant (SOT) recipients is ≈15-fold higher than in the general immunocompetent population,^1^ and SOT patients with COVID-19 appear to be at higher risk of severe outcomes on the basis of their chronically immunosuppressed state and underlying medical comorbidities.^1,3,4^ In 1013 transplant recipients from across Europe, 28-d mortality risk in SOT was ≈20% to 30%^5^ (compared with 0.8%–2% in the general population).^6^ However, whether SOT patients have worse COVID-19–attributable mortality than demographic and comorbidity-matched non-SOT patients after hospitalization remains to be seen, with some earlier studies suggesting no difference once hospitalized.^7^ The largest study to date in SOT patients with COVID-19 is a meta-analysis of 74 published studies from March 2020 to January 2021, including 5559 kidney transplant recipients (51% from Europe and 34% from the United States).^8^ Although there was significant heterogeneity in reported outcomes, AKI occurred in ≈50% of patients and 23% of kidney transplant recipients died; a mortality rate ≈4× to 10× higher than age-matched population controls.^8^ Furthermore, in addition to standard COVID-19 risks, SOT patients with COVID-19 are uniquely at risk for graft loss relating to multiorgan failure and cytokine storm or to graft rejection secondary to immunosuppression withdrawal if critically ill.^9-11^

While predictors of testing positive for COVID-19 have been described in the general non-SOT population, characteristics associated with COVID-19 diagnosis in SOT recipients have not been rigorously explored. There are both biologic and behavioral reasons why certain patients may be at increased risk of acquiring SARS-CoV-2 infection either based on exposure risk or infection after exposure to an inoculum. For example, estrogens are known to be immune stimulating and in the immunocompetent state, females have more robust antiviral immunity than males.^12^ Indeed, earlier literature has demonstrated that in the immunocompetent (non-SOT) population, males are more likely to test positive for COVID-19 than are females.^13^ Whether this relates to sex-based differences in exposure risk or in biologic immunity remains to be seen. Similar behavioral and biologic differences in infection risk after exposure to inoculum may likewise exist for other patient characteristics. Those with underlying comorbid conditions may be more likely to follow public health recommendations to minimize exposure risk and subsequent adverse outcomes from SARS-CoV-2. However, it is known that SARS-CoV-2 enters cells via the angiotensin converting enzyme-2 (ACE2) receptor which may be expressed differentially based on age and underlying comorbidities (chronic obstructive pulmonary disease [COPD], smoking, hypertension, diabetes, and coronary artery disease [CAD]),^14^ and this may have a biologic impact on infection risk following an exposure. In a non-SOT population, a meta-analysis demonstrated hypertension to be the most common comorbidity (30%) in those with COVID-19, followed by diabetes (19%) and CAD (8%).^15^ Common characteristics among hospitalized COVID-19 patients include hypertension (56.6%), as well as obesity (41.7%) and diabetes (33.8%).^16^ Finally, in the general population, advanced age has also been associated with an increased susceptibility to COVID-19 disease (defined as an increased probability of SARS-CoV-2 infection upon contact with an infected individual).^17^ In SOT patients, non-White ethnicity, obesity, asthma/COPD, and diabetes have been associated with COVID-19 disease.^1,10^ However, whether this simply reflects SOT patient demographics or true differences between SOT patients who test positive or negative for COVID-19 has not been examined.

The National COVID Cohort Collaborative (N3C) is the largest database on COVID-19 in the United States. As of July 30, 2021 (release 40), N3C contains longitudinal Electronic Health Record data on >2.1 million SARS-CoV-2 infected patients and >4.3 million uninfected controls from 57 data providers. It consists of a centralized, secure Enclave to store and study vast amounts of medical record data from people diagnosed with COVID-19 across the United States.^18,19^ N3C aims to transform clinical information into the knowledge urgently needed to study COVID-19, including risk factors that indicate better or worse outcomes of the disease. Therefore, using the N3C, in a cohort of SOT patients tested for COVID-19, our objectives were to identify significant differences between those who tested negative and positive, and to determine outcomes following COVID-19 diagnosis in a SOT population.

## MATERIALS AND METHODS

N3C includes a broad category of patients with limited inclusion criteria for incoming data; specifically need for COVID-19 testing for both inpatient and outpatient encounters.^20^ The incoming data comes from 4 primary data models—OMOP, PCORnet, TriNetX, and ACT—harmonized into the OMOP 5.3.1 data model and made available within a secure Enclave for analysis at the patient and encounter level.^18^

### Design

We conducted a cohort study of adult SOT patients (>18 years of age) in the United States who were tested for COVID-19 (either a positive or negative result, COVID+ and COVID−, respectively) between January 1, 2020, and November 20, 2020, identified using the N3C Enclave. The N3C Enclave was developed to facilitate analysis of patient-level data across the United States for multiple conditions, consisting of weekly electronic medical record (EMR) data extraction and transmission into a federally secured platform. This database includes EMR data from 57 US academic medical centers harmonized into an accessible analytical database (see Acknowledgments), including information on >2.1 million COVID+ patients. Any patient tested for COVID-19 (including COVID− individuals) was included in the N3C Enclave before the November 20, 2020, cohort extraction date. Beginning with all releases after the N3C computable phenotype 3.0, however,^21^ all uninfected (COVID−) controls have all been shown to impact ACE2 receptor expression (based on age, sex, and race) in a 2:1 uninfected:infected ratio at each data provider. Because of this matching process, all data released into the Enclave after release 13 (November 20, 2021) are difficult to use for studies requiring a true control arm; after this date, it was not possible to compare age, race, or sex differences between COVID+ and COVID− patients. As a result, we restrict to data collected as of November 20 (release 13), which represents >2 million total patients, including 292 thousand SARS-CoV-2 infected patients from 34 data providers.

### Analysis

Descriptive statistics were used to report baseline characteristics for all SOT patients stratified by whether they tested positive or negative for COVID-19. Counts and percentages were used to describe categorical variables.

### Data Collection

Patient factors that may be associated with increased likelihood of testing positive for COVID-19 were determined a prioiri and included patient demographics, such as age, race, and sex, type of organ transplant (kidney, liver, heart, lung, and other/unknown), and comorbidities (chronic kidney disease [CKD], hypertension, diabetes, COPD/asthma, cancer, CAD, congestive heart failure [CHF], peripheral vascular disease, liver disease, and obesity [body mass index (BMI) > 30 kg/m^2^]). Given large amounts of missingness for BMI (>40%), an indicator was created for missing BMI and included as an adjustment variable in multivariate analyses. We also collected information on maintenance immunosuppression (prednisone, tacrolimus, cyclosporine, and mycophenolate mofetil) and induction agent (antithymocyte globulin [ATG] and basiliximab) utilized during a 90-d look-back period before COVID-19 testing date. Thus, for choice of induction agent, we only had data available for those transplanted in the 90 d before COVID-19 test date. Concept sets defining all standardized vocabulary used for medications, labs, procedures, and outcomes are available on the project Github repository.^22^

### Primary Analysis

In our primary analysis, among those tested, the odds of receiving a positive COVID-19 result was determined using univariable logistic regression for each patient variable. Any patient factor significantly associated with a positive COVID-19 test result at a *P* value of <0.05 was then incorporated into a multivariable logistic regression model to determine the adjusted odds ratio of testing positive for COVID-19.

### Secondary Analysis

In a secondary analysis, we determined the proportion of patients experiencing an outcome in the 90 d after being tested for COVID-19 (among COVID+ and COVID−). Specifically, we examined outcomes including the development of severe COVID-19 requiring hospitalization (in those testing positive), MACE (defined as acute myocardial infarction, angina, stent occlusion/thrombosis, stroke, transient ischemic attack, CHF, or death from any cause), AKI, major adverse renal or cardiac event (MARCE), defined as either a MACE or AKI event, mortality, graft rejection, and graft failure. Because of small event numbers and a requirement of N3C to obscure counts of <20 individuals, we could not report 90-d rejection or mortality rates.

Among all patients tested for COVID-19, we used univariable logistic regression to determine patient variables (including the results of COVID-19 testing) associated with the odds of developing each outcome ([1] MACE, [2] AKI, [3] MARCE, [4] mortality, [5] graft rejection, and [6] graft failure). The odds ratio for each variable for the outcome of MACE, AKI, MARCE, mortality, graft rejection, or graft failure was compared with the odds ratio associated with testing positive for COVID-19. We did not include measures of COVID-19 severity in our regression analysis (eg, hospitalization, need for mechanical ventilation, and extracorporeal membrane oxygenation) because the primary objective was to determine whether COVID-19 (including all potential downstream complications) was associated with the above adverse outcomes. To include COVID-19 complications which may lie on the causal pathway between diagnosis and outcome would attenuate any signal associated with COVID-19 risk.

National Institute of Health’s N3C Data Utilization Request Approval committee approved the data utilization request of this project (RP-CA3365). Each author’s home institution executed Data Use Agreements for participation in N3C. All research team members relied upon Data Use Agreements executed between their home institutions and National Center for Advancing Translational Sciences for access to N3C. Our study protocol was approved by the N3C Data Access and Ethics Committee before analysis. NACTS reviewed all data elements before extraction. All statistical analyses were performed using R.

## RESULTS

At the time of the analysis, 34 sites accounted for over 2 million patients in the Enclave, of whom 292 226 were COVID+. We identified 18 121 SOT patients, of whom 1925 were COVID+ (10.6%) with a median follow-up time of 76 d (0–90 d), Figure S1, SDC, http://links.lww.com/TXD/A372. Demographics for COVID+ versus COVID− SOT patients are shown in Table 1. Compared with COVID− SOT patients, COVID+ SOT patients were slightly younger (mean 54.4 versus 56.1 y, *P* < 0.001) and less likely to be White race (40.3% of COVID+ SOT were White versus 60.3% of COVID−, *P* < 0.001). Those who were COVID+ were significantly more likely to have had a kidney transplant relative to other organ transplant type (72.7% of COVID+ versus 58.1% of COVID− SOT had a kidney transplant, *P* < 0.001). Conversely, lung and liver transplant recipients made up a smaller proportion of those patients testing positive for COVID-19 than those testing negative. Hypertension, diabetes, CAD, peripheral vascular disease, and CKD were common comorbidities in all SOT but significantly more common in those who were COVID+. Further analysis in more recent cohorts was limited by the inclusion of COVID− individuals matched by age, race, and sex.

**TABLE 1.** Patient demographics among solid organ transplant recipients with and without COVID-19

Variable	COVID+ (n/%), N = 1925 (10.6%)	COVID− (n/%), N = 16 196 (89.4%)	*P*
Sex			
Male	1156 (60.1)	9521 (58.8)	0.286
Female	769 (39.9)	6675 (41.2)	
Age			<0.001^*a*^
18–45 y	499 (25.9)	3629 (22.4)	
45–65 y	951 (49.4)	7900 (48.8)	
>65 y	475 (24.7)	4667 (28.8)	
Race			<0.001^*a*^
White	776 (40.3)	9768 (60.3)	
Black or African American	620 (32.2)	3376 (20.8)	
Hispanic or Latino	324 (16.8)	1689 (10.4)	
Other/unknown	205 (10.6)	1363 (8.4)	
Organ type			<0.001^*a*^
Kidney	1399 (72.7)	9415 (58.1)	
Liver	327 (17.0)	4041 (25.0)	
Lung	139 (7.2)	2342 (14.5)	
Other/unknown	243 (12.6)	2178 (13.4)	
Comorbidities			
CKD	1444 (75.0)	9399 (58.0)	<0.001^*a*^
Hypertension	1669 (86.7)	10889 (67.2)	<0.001^*a*^
Diabetes	1198 (62.2)	7851 (48.5)	<0.001^*a*^
Asthma	335 (17.4)	2495 (15.4)	0.024^*a*^
Cancer	459 (23.8)	3081 (19.0)	<0.001^*a*^
Coronary artery disease	1311 (68.1)	9016 (55.7)	<0.001^*a*^
Congestive heart failure	590 (30.6)	4068 (25.1)	<0.001^*a*^
Peripheral vascular disease	523 (27.2)	2992 (18.5)	<0.001^*a*^
Liver disease	556 (28.9)	4842 (29.9)	0.372
Obesity	487 (25.3)	4000 (24.7)	0.001^*a*^
Obesity missing	677 (35.2)	5141 (31.7)	0.001^*a*^
Immunosuppression			
Prednisone	1332 (69.2)	10230 (63.2)	<0.001^*a*^
Tacrolimus	1415 (73.5)	10271 (63.4)	<0.001^*a*^
Cyclosporine	167 (8.7)	1439 (8.9)	0.792
MMF	1319 (68.5)	9535 (58.9)	<0.001^*a*^
ATG induction	199 (10.3)	1123 (6.9)	<0.001^*a*^
Basiliximab induction	63 (3.3)	976 (6.0)	<0.001^*a*^

^*a*^ Statistically significant at a *P* < 0.05.

ATG, antithymocyte globulin; CKD, chronic kidney disease; COVID−, negative result from COVID-19 testing; COVID+, positive result from COVID-19 testing; COVID-19, coronavirus disease 2019; MMF, mycophenolate mofetil.

Results of univariable analysis examining predictors of testing positive for COVID-19 are shown in Table 2. Among those tested, older age was negatively associated with testing positive (odds ratio [OR] 0.74; 95% CI, 0.65-0.85 for those >65 y of age versus those aged 18–45 y). When tested, those of non-White ethnicity and recipients of kidney versus other organ transplants were more likely to have a positive test result, as were patients with any comorbidity other than liver disease. Among those with SOT, prednisone, tacrolimus, and mycophenolate mofetil use were each associated with an increased odds of testing positive for COVID-19. In multivariable analysis, age over 65, liver or lung transplant, and basiliximab induction were independently associated with a lower likelihood of testing positive for COVID-19, whereas a positive test result was more common in those of non-White race, those with hypertension, diabetes, peripheral vascular disease, and tacrolimus maintenance immunosuppressive therapy, Table 2.

**TABLE 2.** Univariable and multivariable OR and 95% CI for a positive COVID-19 test result among solid organ transplant patients who were tested

Variable	Univariable OR (95% CI)	Multivariable OR (95% CI)
Sex		
Male	Ref	–
Female	0.95 (0.86-1.04)	
Age		
18–45 y	Ref	Ref
45–65 y	0.88 (0.78-0.98)	0.99 (0.98-1.00)
>65 y	0.74 (0.65-0.85)	0.97 (0.96-0.99)^*a*^
Race		
White	Ref	Ref
Black or African American	2.31 (2.07-2.59)	1.06 (1.05-1.07)^*a*^
Hispanic or Latino	2.41 (2.1-2.78)	1.07 (1.06-1.09)^*a*^
Other/unknown	1.89 (1.61-2.23)	1.04 (1.03-1.06)^*a*^
Organ type		
Kidney	1.92 (1.72-2.13)	1.02 (1.00-1.03)
Liver	0.62 (0.54-0.70)	0.97 (0.96-0.98)^*a*^
Lung	0.46 (0.39-0.55)	0.97 (0.95-0.99)^*a*^
Other/unknown	0.93 (0.81-1.07)	–
Comorbidities		
CKD	2.17 (1.95-2.42)	1.01 (1.00-1.02)
Hypertension	3.18 (2.77-3.64)	1.05 (1.04-1.06)^*a*^
Diabetes	1.75 (1.59 -1.93)	1.02 (1.01-1.03)^*a*^
Asthma	1.16 (1.02-1.31)	1.01 (1.00-1.02)
Cancer	1.33 (1.19-1.46)	1.01 (1.00-1.02)
Coronary artery disease	1.70 (1.54-1.88)	1.01 (1.00-1.03)
Congestive heart failure	1.32 (1.19-1.46)	0.99 (0.98-1.00)
Peripheral vascular disease	1.65 (1.48-1.83)	1.02 (1.01-1.03)^*a*^
Liver disease	0.95 (0.86-1.06)	–
Obesity	1.13 (1.00-1.27)	1.00 (0.99-1.02)
Obesity missing	1.22 (1.09-1.36)	1.01 (1.00-1.02)
Immunosuppression		
Prednisone	1.31 (1.18-1.45)	0.99 (0.98-1.01)
Tacrolimus	1.60 (1.44-1.78)	1.03 (1.02-1.04)^*a*^
Cyclosporine	0.97 (0.82-1.15)	–
MMF	1.52 (1.37-1.68)	1.01 (1.00-1.02)
ATG induction	1.55 (1.32-1.81)	1.00 (0.98-1.02)
Basiliximab induction	0.53 (0.41-0.68)	0.95 (0.93-0.97)^*a*^

^*a*^ Statistically significant at a *P* < 0.05.

ATG, antithymocyte globulin; CI, confidence interval; CKD, chronic kidney disease; COVID-19, coronavirus disease 2019; MMF, mycophenolate mofetil; OR, odds ratio.

**FIGURE 1.**
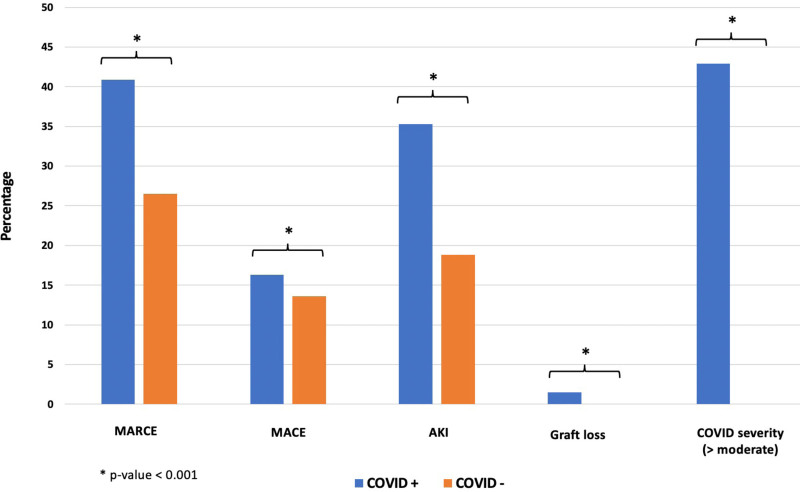
Outcomes after coronavirus disease 2019 (COVID-19) diagnosis in solid organ transplant recipients. AKI, acute kidney injury; COVID−, negative result from COVID-19 testing; COVID+, positive result from COVID-19 testing; Covid-severity >moderate, need for hospitalization, ventilation, ECMO, or death from COVID-19; MACE, major adverse cardiac event; MARCE, major adverse renal or cardiac event.

Ninety-day outcomes in those who tested positive and negative for COVID-19 are shown in Figure 1. In COVID+, 42.9% were hospitalized for a median of 5 d (Q1 2, Q3 10 d). Compared with 26.5% of SOT patients who experienced MARCE in the 90 d following a negative COVID-19 test, 40.9% of SOT patients who tested positive for COVID-19 experienced MARCE. This was driven primarily by an increased incidence of AKI (35.3% of patients with COVID-19 developed AKI, compared with 18.8% of patients testing negative). Graft failure occurred in 1.5% of COVID+ patients. All outcomes were significantly different in COVID+ and COVID− patients at a *P* value of <0.001. Less than 20 COVID− patients experienced graft failure in the 90 d after testing and fewer than 20 SOT patients testing either positive or negative for COVID-19 rejected their organs or died, and thus, the rates for these outcomes could not be displayed.

The odds of developing an outcome in the 90 d after a positive COVID-19 test (relative to those testing negative) are shown in Table 3. Testing positive for COVID-19 was significantly associated with an increased risk of MARCE (OR, 1.92; 95% CI, 1.75-2.12), MACE (OR, 1.23, 95% CI, 1.09-1.40), AKI (OR, 2.35, 95% CI, 2.13-2.61), death (OR, 8.43, 95% CI, 2.11-33.73), rejection (OR, 31.8, 95% CI, 10.5-95.9), and graft loss (OR, 79.7, 95% CI, 24.2-262.31).

**TABLE 3.** OR and 95% CI of 90-d outcomes associated with COVID-19 test positivity

Outcome	COVID+ (n/%)	COVID− (n/%)	OR (95% CI)
MARCE	788 (40.9)	4290 (26.5)	1.92 (1.75-2.12)
MACE	314 (16.3)	2208 (13.6)	1.23 (1.09-1.40)
Mortality	<20^*a*^	<20^*a*^	8.43 (2.11-33.73)
Covid-severity (>moderate)	826 (42.9)	–	–
AKI	680 (35.3)	3050 (18.8)	2.35 (2.13-2.61)
Rejection	<20^*a*^	<20^*a*^	31.79 (10.54-95.88)
Graft loss	28 (1.5)	<20^*a*^	79.67 (24.20-262.31)

^*a*^Event counts of <20 are not reported in alignment with N3C privacy requirements.

AKI, acute kidney injury; CI, confidence interval; COVID−, negative result from COVID-19 testing; COVID+, positive result from COVID-19 testing; COVID-19, coronavirus disease 2019; Covid-severity >moderate, need for hospitalization, ventilation, ECMO, or death from COVID-19; MACE, major adverse cardiac event; MARCE, major adverse renal or cardiac event; N3C, National COVID Cohort Collaborative; OR, odds ratio.

Among all SOT patients tested (COVID+ and COVID−), predictors of each outcome (MARCE, MACE, AKI, mortality, rejection, and graft loss) are shown in Table 4. Compared with all other organ types, kidney transplant recipients had the highest risk of AKI (OR, 1.26, 95% CI, 1.16-1.35) and graft loss (OR, 6.32, 95% CI, 1.92-20.80). Lung and liver transplant recipients had significantly lower odds of MARCE and MACE, and lung recipients also had a lower odds of AKI after being tested for COVID-19, compared with other allograft types.

**TABLE 4.** OR and 95% CI for 90-d outcomes after testing for COVID-19 (both positive and negative test results)

Variable	MARCE	MACE	AKI	Rejection	Death	Graft loss
COVID-19+ (vs −)	1.92 (1.75-2.12)^*a*^	1.23 (1.09-1.40)^*a*^	2.35 (2.13-2.61)^*a*^	31.79 (10.54-95.88)^*a*^	8.43 (2.11-33.73)^*a*^	79.67 (24.2-262.31)^*a*^
Female sex (vs male)	0.88 (0.82-0.94)^*a*^	0.83 (0.76-0.91)^*a*^	0.92 (0.85-0.99)^*a*^	0.51 (0.18-1.42)	0.86 (0.21-3.60)	0.50 (0.22-1.11)
Age						
18–45 y	Ref	Ref	Ref	Ref	Ref	Ref
45–65 y	1.21 (1.11-1.32)^*a*^	1.59 (1.40-1.80)^*a*^^,^^*b*^	1.11 (1.01-1.22)^*a*^	1.21 (0.43-3.40)	2.33 (0.27-19.97)	1.20 (0.50-2.87)
>65 y	1.42 (1.29-1.56)^*a*^	2.47 (2.18-2.81)^*a*^^,^^*b*^	1.13 (1.02-1.25)^*a*^	0.16 (0.02-1.37)	1.61 (0.15-17.72)	0.69 (0.23-2.05)
Race						
White	Ref	Ref	Ref	Ref	Ref	Ref
Black or African American	1.66 (1.53-1.79)^*a*^	1.58 (1.43-1.74)^*a*^^,^^*b*^	1.55 (1.43-1.70)^*a*^	1.89 (0.60-5.95)	0.53 (0.06-4.52)	2.88 (1.27-6.54)^*a*^
Hispanic or Latino	1.16 (1.04-1.29)^*a*^	0.87 (0.75-1.02)	1.29 (1.15-1.45)^*a*^	3.75 (1.19-11.82)^*a*^	1.05 (0.12-8.97)	1.91 (0.61-5.99)
Other/unknown	1.22 (1.08-1.37)^*a*^	1.00 (0.85-1.17)	1.26 (1.10-1.43)^*a*^	1.92 (0.40-9.26)	1.35 (0.16-11.52)	2.45 (0.78-7.70)
Organ type						
Kidney	1.17 (1.10-1.25)^*a*^	1.03 (0.94-1.12)	1.26 (1.16-1.35)^*a*^	1.16 (0.46-2.94)	1.13 (0.27-4.71)	6.32 (1.92-20.80)^*a*^
Liver	0.82 (0.76-0.89)^*a*^	0.57 (0.51-0.63)^*a*^	0.96 (0.88-1.05)	0.59 (0.17-2.03)	1.05 (0.21-5.20)	0.34 (0.10-1.11)
Lung	0.71 (0.64-0.78)^*a*^	0.64 (0.55-0.73)^*a*^	0.80 (0.72-0.90)^*a*^	0.74 (0.18-3.21)	0.90 (0.11-7.32)	–
Other/unknown	1.59 (1.46-1.74)^*a*^	2.8 (2.53-3.10)^*a*^^,^^*b*^	1.00 (0.90-1.11)	1.73 (0.57-5.22)	0.93 (0.11-7.53)	0.69 (0.21-2.21)
Comorbidities						
CKD	2.01 (1.87-2.15)^*a*^^,^^*b*^	1.66 (1.52-1.82)^*a*^^,^^*b*^	2.18 (2.01-2.36)^*a*^	1.88 (0.68-5.22)	1.12 (0.27-4.68)	20.19 (2.75-148.06)^*a*^
Hypertension	1.73 (1.60-1.86)^*a*^	1.38 (1.25-1.51)^*a*^^,^^*b*^	1.80 (1.65-1.96)^*a*^	3.77 (0.87-16.32)	1.33 (0.27-6.59)	–
Diabetes	1.78 (1.67-1.90)^*a*^	1.82 (1.67-1.99)^*a*^^,^^*b*^	1.75 (1.63-1.88)^*a*^	2.81 (1.01-7.80)^*a*^	3.01 (0.61-14.91)	1.22 (0.60-2.47)
Asthma	1.51 (1.39-1.65)^*a*^	1.72 (1.55-1.91)^*a*^^,^^*b*^	1.33 (1.21-1.46)^*a*^	3.94 (1.58-9.80)^*a*^	0.77 (0.09-6.27)	0.80 (0.28-2.29)
Cancer	1.38 (1.28-1.49)^*a*^	1.15 (1.03-1.27)^*a*^	1.46 (1.34-1.59)^*a*^	0.23 (0.03-1.71)	0.59 (0.07-4.78)	1.20 (0.52-2.79)
Coronary artery disease	2.47 (2.30-2.65)^*a*^^,^^b^*	3.95 (3.56-4.40)^*a*^^,^^*b*^	1.90 (1.76-2.05)^*a*^	1.64 (0.62-4.31)	1.26 (0.30-5.27)	1.59 (0.75-3.37)
Congestive heart failure	3.87 (3.60-4.15)^*a*^^,^^*b*^	13.55 (12.28-14.95)^*a*^^,^^*b*^	1.83 (1.69-1.97)^*a*^	1.33 (0.51-3.51)	2.89 (0.72-11.57)	1.38 (0.65-2.93)
Peripheral vascular disease	1.78 (1.65-1.93)^*a*^	2.07 (1.89-2.28)^*a*^^,^^*b*^	1.62 (1.49-1.77)^*a*^	1.48 (0.53-4.12)	1.39 (0.28-6.87)	1.98 (0.93-4.21)
Liver disease	1.20 (1.12-1.28)^*a*^	0.95 (0.87-1.04)	1.33 (1.23-1.43)^*a*^	0.63 (0.21-1.89)	1.41 (0.34-5.92)	0.69 (0.30-1.60)
Obesity	1.09 (1.01-1.18)^*a*^	1.26 (1.14-1.39)^*a*^^,^^*b*^	1.01 (0.93-1.11)	1.09 (0.36-3.33)	3.48 (0.32-38.44)	0.70 (0.27-1.80)
Obesity missing	0.92 (0.86-1.00)	0.93 (0.84-1.03)	0.99 (0.91-1.07)	1.01 (0.35-2.91)	6.72 (0.79-57.54)	0.90 (0.40-1.99)
Immunosuppression						
Prednisone	0.88 (0.82-0.94)^*a*^	0.85 (0.78-0.92)^*a*^	0.89 (0.83-0.96)^*a*^	1.59 (0.57-4.41)	3.97 (0.49-32.3)	1.19 (0.56-2.53)
Tacrolimus	0.73 (0.68-0.78)^*a*^	0.63 (0.58-0.69)^*a*^	0.80 (0.74-0.86)^*a*^	4.69 (1.08-20.28)^*a*^	0.33 (0.08-1.38)	0.76 (0.37-1.56)
Cyclosporine	1.16 (1.04-1.30)^*a*^	1.11 (0.96-1.29)	1.22 (1.08-1.38)^*a*^	0.57 (0.08-4.28)	3.43 (0.69-17.01)	1.10 (0.33-3.63)
MMF	0.79 (0.74-0.85)^*a*^	0.70 (0.64-0.76)^*a*^	0.84 (0.79-0.91)^*a*^	1.88 (0.68-5.21)	2.01 (0.41-9.96)	0.81 (0.40-1.65)
ATG induction	1.20 (1.06-1.35)^*a*^	0.80 (0.68-0.96)^*a*^	1.26 (1.10-1.44)^*a*^	0.71 (0.09-5.29)	1.82 (0.22-14.77)	1.36 (0.41-4.49)
Basiliximab induction	0.84 (0.73-0.98)^*a*^	0.60 (0.48-0.75)^*a*^	0.93 (0.80-1.09)	NA	NA	0.55 (0.07-4.02)

^*a*^Statistically significant at *P* < 0.05.

^*b*^Stronger (significant) predictors of the specific outcome than a positive COVID-19 test result.

AKI, acute kidney injury; ATG, antithymocyte globulin; CI, confidence interval; CKD, chronic kidney disease; COVID-19, coronavirus disease 2019; MACE, major adverse cardiac event; MARCE, major adverse renal or cardiac event; MMF, mycophenolate mofetil; NA, not available; OR, odds ratio.

A positive COVID-19 test result was a stronger predictor of each outcome (MARCE, MACE, AKI, mortality, rejection, and graft loss) than most other patient variables, Table 4. The only variables more strongly associated with MARCE were a history of CKD, CAD, or CHF. Testing positive for COVID-19 was the strongest predictor of AKI (more so even than a preexisting diagnosis of CKD), as well as death, rejection, and graft failure.

## DISCUSSION

Our analysis of patient characteristics associated with testing positive for COVID-19 in SOT recipients includes 1925 COVID+ SOT from multiple US medical centers and represents the first analysis using real-world EMR data examining the impact of the COVID-19 pandemic in transplantation. In this study, we compared patient characteristics and comorbidities in those who tested positive versus negative for COVID-19 and demonstrated important differences between the 2 groups. Earlier studies of COVID-19 in SOT examine results only in those with a confirmed diagnosis of COVID-19^8,23-25^ which is an important but different question. By excluding those who test negative, it is impossible to determine factors that are more likely to associate with a COVID-19 diagnosis. Therefore, our study is novel. We show that compared with COVID− patients, COVID+ SOT recipients were younger, less likely to be White race, and more likely to have had a kidney transplant than another organ. They also had greater levels of comorbidity, with a higher prevalence of hypertension, diabetes, CAD, peripheral vascular disease, and CKD. Despite the increased risk associated with male sex in the general population,^3,26^ male SOT recipients were not more likely to test positive for COVID-19. Likewise, in the general population, older age is a risk for SARS-CoV-2 infection.^17^ However, in our population, we saw the opposite in that SOT COVID+ recipients were significantly younger than those testing negative. Whether the association between male sex and older age with COVID-19 diagnosis in the general population relates to a biologic vulnerability to viral infection or to differences in behaviors and lifestyles (potentially more travel and contact patterns in males or older individuals leading to more virus exposure) that might put these patients at increased risk of acquiring COVID-19 remains to be seen. We may speculate that older male SOT patients are not at increased risk for acquiring COVID-19 because of better adherence to social distancing and use of personal protective equipment thereby mitigating any behavioral differences observed in the general population. It is also possible that given concerns regarding amplified risk with these demographics, more asymptomatic older individuals and males were tested leading to an influx of negative screening results. The same may have been the case for SOT recipients with comorbidities; with more testing and better adherence to public health recommendations in those at higher risk. Finally, we demonstrate that, not surprisingly, immunosuppression use was associated with an increased risk of SARS-CoV-2 infection. It is known that immunosuppression is a risk for opportunistic and other infections, and there is literature to support that infectious complications likely vary by immunosuppressive agent. For example, in patients being treated for lupus nephritis, a meta-analysis has demonstrated significant differences in infectious risk by immunosuppressive drug; tacrolimus being associated with a lower risk of serious infections compared with cyclophosphamide and prednisone.^27^ Interestingly, in our study, tacrolimus was the only immunosuppressive agent independently associated with testing positive for COVID-19 in multivariable analysis. The significance of this finding requires further investigation.

In the 90 d following COVID-19 diagnosis, we identify that 42.9% of SOT recipients required hospitalization, compared with the hospitalization rate for COVID-19 in the general public which has been shown to be 14% to 46%.^28,29^ Importantly, we also show an increased 90-d incidence of AKI following COVID-19 diagnosis. COVID-19 has been associated with an increased risk of AKI in the general population through potentially coexisting pathways, including both direct and indirect effects. Direct viral invasion of kidney cells occurs through the ACE2 receptor on endothelial cells and podocytes resulting in endothelial dysfunction and nephritis, inciting a hyperinflammatory response, hypercoagulability, and complement activation.^30,31^ Indirect renal consequences of COVID-19 may relate to hemodynamic instability in severe disease, organ cross-talk, activation of the renin-angiotensin-aldosterone system, rhabdomyolysis and sepsis or acute respiratory distress syndrome-associated AKI.^30,31^

We also demonstrate that in the 3 mo after diagnosis, 16.3% of SOT patients experienced MACE, compared with the annual incidence of MACE in SOT patients without COVID-19 of ≈3.5%–5%.^32^ In our study, however, the risk of MACE in the COVID− cohort was also much higher (13.6%), reflecting a more complex control population who by fact of inclusion, had an indication for COVID-19 testing. The increased MACE risk following COVID-19 diagnosis likely reflects a physiologic response to critical illness, but it has also been proposed that the overwhelming inflammatory response to COVID-19 itself may trigger a cascade of complications culminating in cardiovascular events.^33^ Finally, we demonstrate 90-d incidence rates of graft loss of 1.5%, with very few events in the COVID− groups (leading to large OR and wide CI associated with COVID-19 test positivity). Event rates in the COVID+ group are lower than in earlier studies of hospitalized patients, which likely reflects the fact that our study includes a less sick population, with nearly 50% of our cohort being managed as outpatients (providing a better estimate of the true natural trajectory of COVID-19 disease in all SOT patients not just those sick enough for admission).

Although we do not have information regarding the COVID-19 management strategies employed in these patients, this would likely suggest less adjustments or minimization of immunosuppression were required. The American Society of Transplantation currently recommends providers consider decreasing nonsteroid immunosuppression in sick SOT patients with COVID-19 and without recent episodes of acute rejection, however, the decision to reduce immunosuppression should be individualized and based on the relative risk-benefit of COVID-19 severity and graft rejection.^34^ Conversely, the American Association for the Study of Liver Diseases makes a more formal statement regarding discontinuation of antimetabolite medications and maintenance of calcineurin inhibitors in those with active COVID-19.^35^

While systematic review and meta-analysis have yielded sizable COVID+ SOT study populations,^8^ the largest COVID+ SOT cohort study to date includes just over 1000 transplant recipients and focuses on outcomes in SOT recipients with COVID-19, rather than predictors of testing positive for COVID-19.^5^ Our current study is the largest to date to explore patient characteristics associated with a diagnosis of COVID-19 in a national cohort of SOT. This information is valuable in that it may help identify patients who require a lower threshold for COVID-19 screening. We demonstrate similarities to the general population in that hypertension, diabetes, and CAD were all associated with testing positive for COVID-19,^15^ however, in our population, the proportion of patients with these comorbidities was much higher (eg, 69.3% of the entire cohort of SOT patients were hypertensive). Irrespective of whether certain patient characteristics are associated with an increased behavioral or biologic risk of acquiring COVID-19, the importance of social distancing and masking cannot be understated as a means of mitigating SARS-CoV-2 exposure and subsequent infection in this immunosuppressed population.

This study has limitations, however. As with all retrospective analyses, there is a risk of miscoding and misclassification of patient covariates or outcomes. Importantly, in the general population, there is a bias in who ultimately undergoes testing for COVID-19 with males, Black ethnicity, social disadvantage (based on education, housing, and income metrics), smoking history, obesity, and comorbid patients being tested more frequently.^36^ We were not able to account for potential differential testing bias, however, we were able to identify significant differences in test positivity among those tested. Additionally, the primary analysis examines predictors of COVID-19 test positivity, however, it is known that there are limitations to test accuracy and false positives and negatives exist in varying frequencies depending on the test utilized. Reverse transcriptase polymerase chain reaction assay of nasopharyngeal respiratory secretions is the current gold standard for COVID-19 diagnosis.^37^ However, this has been associated with a relatively high false-negative rate.^38,39^ Alternative diagnostic tests include serologic antibody testing for SARS-CoV-2 antibodies and viral antigen testing.^40^ However, the reliability of antibody testing in immunosuppressed SOT patients has been questioned, and viral antigen testing is known to be limited by reduced sensitivity and high false-negative rates.^40^ However, we did not have information on what particular test was used for diagnosis. Importantly, neither did we have information on the indication for testing or presenting symptoms. A meaningful risk prediction model for COVID-19 diagnosis in SOT recipients would need to incorporate patient symptoms in addition to underlying demographics and comorbidity burden. Patients testing negative for COVID-19 would have had an indication for testing (whether asymptomatic screening at the time of a routine clinic visit, or emergency room presentation with respiratory compromise), and thus because of potential confounding by indication, the risk of 90-d outcomes is inevitably higher in this population than in those not requiring testing. Because of limits with data availability, we do not have robust historical data on induction agent utilized at the time of transplant, as medications were only captured in a look-back window of 90 d. Therefore, it is unlikely we would have induction data on those transplanted >90 d before COVID-19 testing date. Despite this limitation, we examined the potential risk associated with ATG versus basiliximab in those with available data, and show that, with the caveat regarding missing data in those farther posttransplant, Basiliximab induction appears to be lower risk than ATG (as might be expected). Another important limitation is that we only had mortality outcome data as part of the MACE composite, not mortality in isolation. Because of very low 90-d rejection and death rates (<20 individual patients) in both the COVID+ and COVID− groups, and concerns regarding patient privacy, we were not able to present isolated rejection or mortality data, which are certainly important metrics. Nor could we present graft loss rates in those who tested negative for COVID. Earlier literature has demonstrated a 20% to 32% 28-d mortality rate in SOT patients with COVID-19.^4,41^ However, again these studies are restricted to SOT patients who are hospitalized with COVID-19 and thus, represent a sicker cohort. Finally, although our study includes both inpatients and outpatients who had testing for COVID-19, many infections are asymptomatic and these patients would not have sought testing unless required for hospital operating procedures and would remain undiagnosed.

### Conclusions

In this study, we examine patient factors associated with having a positive result when SOT recipients are tested for COVID-19. This study includes 30 times more SOT patients than the next largest study examining predictors of a positive COVID-19 test in this population. We show that those with a kidney transplant are the highest risk for acquiring COVID-19, and comorbidities are common in all SOT patients but are even more common in those testing positive for COVID-19. SOT patients who test positive for COVID-19 are at a high risk for hospitalization, MARCE, and AKI. This information is important to risk stratify patients who might require testing for COVID-19.

## ACKNOWLEDGMENTS

The National Institute of Health’s National COVID Cohort Collaborative (N3C) Data Utilization Request Approval committee approved the data utilization request of this project (RP-CA3365). Each author’s home institution executed Data Use Agreements for participation in N3C. All research team members relied upon Data Use Agreements executed between their home institutions and National Center for Advancing Translational Sciences for access to N3C. Our study protocol was approved by the N3C Data Access Committee before analysis. NACTS reviewed all data elements before extraction. This research was possible because of the patients whose information is included within the data from participating organizations (https://ncats.nih.gov/n3c/resources/data-contribution/data-transfer-agreement-signatories) and scientists who have contributed to the on-going development of this community resource (https://doi.org/10.1093/jamia/ocaa196). N3C Principal Investigators: Melissa A. Haendel,* Christopher G. Chute,* Kenneth R. Gersing, and Anita Walden. Workstream, subgroup and administrative leaders: Melissa A. Haendel,* Tellen D. Bennett, Christopher G. Chute, David A. Eichmann, Justin Guinney, Warren A. Kibbe, Hongfang Liu, Philip R.O. Payne, Emily R. Pfaff, Peter N. Robinson, Joel H. Saltz, Heidi Spratt, Justin Starren, Christine Suver, Adam B. Wilcox, Andrew E. Williams, and Chunlei Wu. Key liaisons at data partner sites. Regulatory staff at data partner sites. Individuals at the sites who are responsible for creating the data sets and submitting data to N3C. Data Ingest and Harmonization Team: Christopher G. Chute,* Emily R. Pfaff,* Davera Gabriel, Stephanie S. Hong, Kristin Kostka, Harold P. Lehmann, Richard A. Moffitt, Michele Morris, Matvey B. Palchuk, Xiaohan Tanner Zhang, and Richard L. Zhu. Phenotype Team (Individuals who create the scripts that the sites use to submit their data, based on the COVID and Long COVID definitions): Emily R. Pfaff,* Benjamin Amor, Mark M. Bissell, Marshall Clark, Andrew T. Girvin, Stephanie S. Hong, Kristin Kostka, Adam M. Lee, Robert T. Miller, Michele Morris, Matvey B. Palchuk, and Kellie M. Walters. Project Management and Operations Team: Anita Walden,* Yooree Chae, Connor Cook, Alexandra Dest, Racquel R. Dietz, Thomas Dillon, Patricia A. Francis, Rafael Fuentes, Alexis Graves, Julie A. McMurry, Andrew J. Neumann, Shawn T. O’Neil, Usman Sheikh, Andréa M. Volz, and Elizabeth Zampino. Partners from National Institutes of Health and other federal agencies: Christopher P. Austin,* Kenneth R. Gersing,* Samuel Bozzette, Mariam Deacy, Nicole Garbarini, Michael G. Kurilla, Sam G. Michael, Joni L. Rutter, and Meredith Temple-O’Connor. Analytics Team (Individuals who build the Enclave infrastructure, help create codesets, variables, and help Domain Teams and project teams with their data sets): Benjamin Amor,* Mark M. Bissell, Katie Rebecca Bradwell, Andrew T. Girvin, Amin Manna, and Nabeel Qureshi. Publication Committee Management Team: Mary Morrison Saltz,* Christine Suver,* Christopher G. Chute, Melissa A. Haendel, Julie A. McMurry, Andréa M. Volz, and Anita Walden. Publication Committee Review Team: Carolyn Bramante, Jeremy Richard Harper, Wenndy Hernandez, Farrukh M. Koraishy, Federico Mariona, Saidulu Mattapally, Amit Saha, and Satyanarayana Vedula. Data partners with released data (50). Stony Brook University—U24TR002306. University of Oklahoma Health Sciences Center—U54GM104938: Oklahoma Clinical and Translational Science Institute. West Virginia University—U54GM104942: West Virginia Clinical and Translational Science Institute. University of Mississippi Medical Center—U54GM115428: Mississippi Center for Clinical and Translational Research. University of Nebraska Medical Center—U54GM115458: Great Plains IDeA-Clinical & Translational Research. Maine Medical Center—U54GM115516: Northern New England Clinical & Translational Research Network. Wake Forest University Health Sciences—UL1TR001420: Wake Forest Clinical and Translational Science Institute. Northwestern University at Chicago—UL1TR001422: Northwestern University Clinical and Translational Science Institute. University of Cincinnati—UL1TR001425: Center for Clinical and Translational Science and Training. The University of Texas Medical Branch at Galveston—UL1TR001439: The Institute for Translational Sciences. Medical University of South Carolina—UL1TR001450: South Carolina Clinical & Translational Research Institute. University of Massachusetts Medical School Worcester—UL1TR001453: The UMass Center for Clinical and Translational Science. University of Southern California—UL1TR001855: The Southern California Clinical and Translational Science Institute. Columbia University Irving Medical Center—UL1TR001873: Irving Institute for Clinical and Translational Research. George Washington Children’s Research Institute—UL1TR001876: Clinical and Translational Science Institute at Children’s National. University of Kentucky—UL1TR001998: UK Center for Clinical and Translational Science. University of Rochester—UL1TR002001: UR Clinical & Translational Science Institute. University of Illinois at Chicago—UL1TR002003: UIC Center for Clinical and Translational Science. Penn State Health Milton S. Hershey Medical Center—UL1TR002014: Penn State Clinical and Translational Science Institute. The University of Michigan at Ann Arbor—UL1TR002240: Michigan Institute for Clinical and Health Research. Vanderbilt University Medical Center—UL1TR002243: Vanderbilt Institute for Clinical and Translational Research. University of Washington—UL1TR002319: Institute of Translational Health Sciences. Washington University in St Louis—UL1TR002345: Institute of Clinical and Translational Sciences. Oregon Health & Science University—UL1TR002369: Oregon Clinical and Translational Research Institute. University of Wisconsin-Madison—UL1TR002373: University of Wisconsin Institute for Clinical and Translational Research. Rush University Medical Center—UL1TR002389: The Institute for Translational Medicine (ITM). The University of Chicago—UL1TR002389: ITM. University of North Carolina at Chapel Hill—UL1TR002489: North Carolina Translational and Clinical Science Institute. University of Minnesota—UL1TR002494: Clinical and Translational Science Institute. Children’s Hospital Colorado—UL1TR002535: Colorado Clinical and Translational Sciences Institute. The University of Iowa—UL1TR002537: Institute for Clinical and Translational Science. The University of Utah—UL1TR002538: Uhealth Center for Clinical and Translational Science. Tufts Medical Center—UL1TR002544: Tufts Clinical and Translational Science Institute. Duke University—UL1TR002553: Duke Clinical and Translational Science Institute. Virginia Commonwealth University—UL1TR002649: C. Kenneth and Dianne Wright Center for Clinical and Translational Research. The Ohio State University—UL1TR002733: Center for Clinical and Translational Science. The University of Miami Leonard M. Miller School of Medicine—UL1TR002736: University of Miami Clinical and Translational Science Institute. University of Virginia—UL1TR003015: iTHRIVL Integrated Translational Health Research Institute of Virginia. Carilion Clinic—UL1TR003015: iTHRIVL Integrated Translational Health Research Institute of Virginia. University of Alabama at Birmingham—UL1TR003096: Center for Clinical and Translational Science. Johns Hopkins University—UL1TR003098: Johns Hopkins Institute for Clinical and Translational Research. University of Arkansas for Medical Sciences—UL1TR003107: University of Arkansas for Medical Sciences Translational Research Institute. Nemours—U54GM104941: Delaware Center for Translational Research ACCEL Program. University Medical Center New Orleans—U54GM104940: Louisiana Clinical and Translational Science Center. University of Colorado Denver, Anschutz Medical Campus—UL1TR002535: Colorado Clinical and Translational Sciences Institute. Mayo Clinic Rochester—UL1TR002377: Mayo Clinic Center for Clinical and Translational Science. Tulane University—UL1TR003096: Center for Clinical and Translational Science. Loyola University Medical Center—UL1TR002389: ITM. Advocate Healthcare Network—UL1TR002389: ITM. OCHIN—INV-018455: Bill and Melinda Gates Foundation grant to Sage Bionetworks. Additional data partners who have signed decision to admit and data release pending (35). The Rockefeller University—UL1TR001866: Center for Clinical and Translational Science. The Scripps Research Institute—UL1TR002550: Scripps Research Translational Institute. University of Texas Health Science Center at San Antonio—UL1TR002645: Institute for Integration of Medicine and Science. The University of Texas Health Science Center at Houston—UL1TR003167: Center for Clinical and Translational Sciences. NorthShore University HealthSystem—UL1TR002389: ITM. Yale New Haven Hospital—UL1TR001863: Yale Center for Clinical Investigation. Emory University—UL1TR002378: Georgia Clinical and Translational Science Alliance. Weill Medical College of Cornell University—UL1TR002384: Weill Cornell Medicine Clinical and Translational Science Center. Montefiore Medical Center—UL1TR002556: Institute for Clinical and Translational Research at Einstein and Montefiore. Medical College of Wisconsin—UL1TR001436: Clinical and Translational Science Institute of Southeast Wisconsin. University of New Mexico Health Sciences Center—UL1TR001449: University of New Mexico Clinical and Translational Science Center. George Washington University—UL1TR001876: Clinical and Translational Science Institute at Children’s National. Stanford University—UL1TR003142: Spectrum: The Stanford Center for Clinical and Translational Research and Education. Regenstrief Institute—UL1TR002529: Indiana Clinical and Translational Science Institute. Cincinnati Children’s Hospital Medical Center—UL1TR001425: Center for Clinical and Translational Science and Training. Boston University Medical Campus—UL1TR001430: Boston University Clinical and Translational Science Institute. The State University of New York at Buffalo—UL1TR001412: Clinical and Translational Science Institute. Aurora Healthcare—UL1TR002373: Wisconsin Network For Health Research. Brown University—U54GM115677: Advance Clinical Translational Research. Rutgers, The State University of New Jersey—UL1TR003017: New Jersey Alliance for Clinical and Translational Science. Loyola University Chicago—UL1TR002389: ITM. #N/A—UL1TR001445: Langone Health’s Clinical and Translational Science Institute. Children’s Hospital of Philadelphia—UL1TR001878: Institute for Translational Medicine and Therapeutics. University of Kansas Medical Center—UL1TR002366: Frontiers: University of Kansas Clinical and Translational Science Institute. Massachusetts General Brigham—UL1TR002541: Harvard Catalyst. Icahn School of Medicine at Mount Sinai—UL1TR001433: ConduITS Institute for Translational Sciences. Ochsner Medical Center—U54GM104940: Louisiana Clinical and Translational Science Center. HonorHealth—None (Voluntary). University of California, Irvine—UL1TR001414: The UC Irvine Institute for Clinical and Translational Science. University of California, San Diego—UL1TR001442: Altman Clinical and Translational Research Institute. University of California, Davis—UL1TR001860: University of California, Davis Health Clinical and Translational Science Center. University of California, San Francisco—UL1TR001872: University of California, San Francisco Clinical and Translational Science Institute. University of California, Los Angeles—UL1TR001881: University of California, Los Angeles Clinical Translational Science Institute. University of Vermont—U54GM115516: Northern New England Clinical & Translational Research Network. Arkansas Children’s Hospital—UL1TR003107: University of Arkansas for Medical Sciences Translational Research Institute.

## Supplementary Material


