# Supplementary material for: COVID-19 in Solid Organ Transplantation: Results of the National COVID Cohort Collaborative
Source: Transplant Direct. 2021 Oct 6;7(11):e775. doi: 10.1097/TXD.0000000000001234 (PMC8500600; doi:10.1097/TXD.0000000000001234)
Supplement: Supplementary file 1 [file txd-7-e775-s001.pdf]

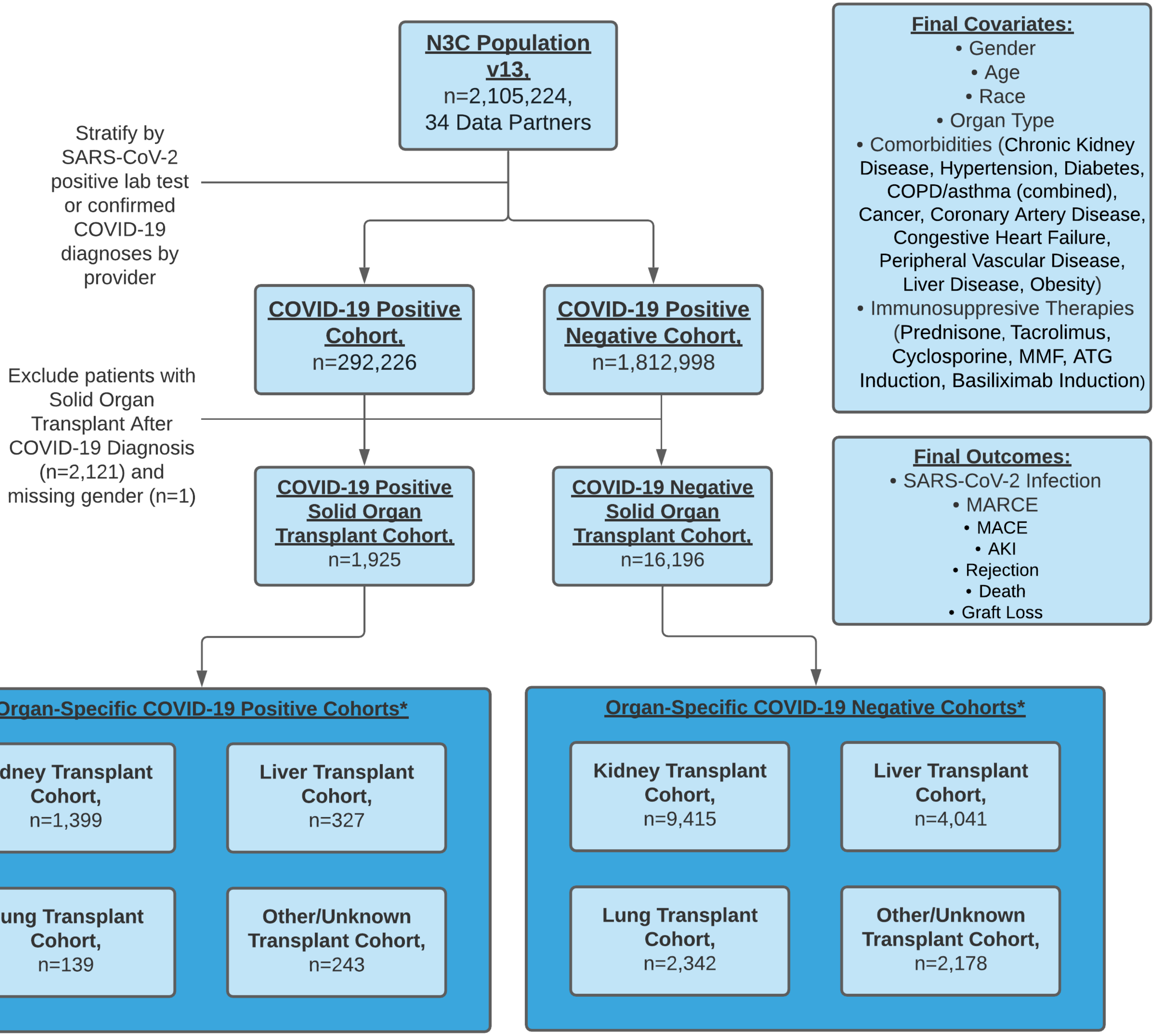

*Concept Set definitions for organ-specific cohorts available here:
https://github.com/National-COVID-Cohort-Collaborative/CS-ISC/blob/main/MARCE/sot_transplant_concept.xlsx
